# A Case of New Delhi Metallo-β-Lactamases (NDM) Citrobacter sedlakii Osteomyelitis Successfully Treated With Ceftazidime-Avibactam and Aztreonam

**DOI:** 10.7759/cureus.28855

**Published:** 2022-09-06

**Authors:** Zachary A Rubnitz, Victoria N Kunkel, Vickie S Baselski, Nathan A Summers

**Affiliations:** 1 Internal Medicine, The University of Tennessee Health Sciences Center, Memphis, USA; 2 Pathology, The University of Tennessee Health Sciences Center, Memphis, USA; 3 Infectious Disease, The University of Tennessee Health Sciences Center, Memphis, USA

**Keywords:** multi-drug resistance (mdr), osteomyelitis, carbapenem resistant enterobacterales (cre), new delhi metallo-beta lactamase (ndm), citrobacter sedlakii

## Abstract

There have been an increase in multi-drug resistant (MDR) organisms causing infections with high mortality and morbidity. Bacteria that carry metallo-β-lactamases (MBLs) are particularly dangerous. Novel antibiotic combinations, such as ceftazidime-avibactam with aztreonam, are in clinical trials for the treatment of MBL-harboring bacteria. We discuss the case of a 39-year-old patient who presented with tibial osteomyelitis growing MBL-producing *Citrobacter sedlakii*. He was successfully treated with ceftazidime-avibactam and aztreonam combination therapy. We discuss the importance of developing new antibiotic regimens for the growing threat of MDR organisms with special consideration of MBL.

## Introduction

Multi-drug resistant (MDR) organisms have become a serious threat across the world and have led to infections with high mortality rates [1]. As we continue to prescribe more advanced antibiotics, bacteria, Enterobacterales in particular, have found ways to develop resistance. One key mechanism of resistance is the production of β-lactamases. These incredibly diverse enzymes have the potential to effectively neutralize nearly every β-lactam. Bacteria may also possess multiple different classes of β-lactamases, leading to resistance to all β-lactam antibiotics [1,2]. Metallo-β-lactamases (MBL) are grouped into Ambler class B and incorporate a zinc molecule into their active site. MBL generally confer resistance to most β-lactams including newer agents like ceftazidime and carbapenems but notably not aztreonam [2]. However, aztreonam is often not effective as monotherapy given that the causative agent can also carry β-lactamases from other classes, such as Ambler class A β-lactamases, which inactivate aztreonam. A novel antibiotic combination now in phase 2 clinical trials is avibactam with aztreonam for MDR organisms with MBL production [3]. This combination allows the aztreonam to kill the bacteria while the avibactam protects the aztreonam from hydrolysis from the class A β-lactamase. This novel combination has been shown to effectively treat MBL organisms both in vitro and in vivo.

## Case presentation

A 30-40-year-old male with a history of tibial fracture complicated by osteomyelitis presented to the clinic with increased pain, purulent drainage, and erythema surrounding his wound. These symptoms had been progressing over the previous week but were not associated with any systemic symptoms. He had initially fractured his right tibia while living in India approximately one year prior to presentation. At that time, he had an intramedullary nail placed and reportedly felt well for the next several months. However, after moving to the United States, he began having increased leg pain and swelling. He was found to have an infected non-union of the right tibia. Orthopedic management included an open reduction and internal fixation with the removal of hardware and insertion of a gentamicin-coated rod. Cultures at that time were polymicrobial including a carbapenem-resistant Enterobacterales (CRE), *Citrobacter sedlakii*, susceptible to eravacycline. He then completed a four-week course of eravacycline parenterally before transitioning to two weeks of omadacycline orally to finish a six-week course.

Unfortunately, there was clinical evidence of recurrent infection with increased purulent drainage from the leg immediately following his initial antibiotic course. He was readmitted and underwent repeat debridement. Intraoperative cultures confirmed relapsed infection with the same CRE *C. sedlakii* isolate, resistant to all initially tested antimicrobials, with limited options available upon additional request (Table 1).

**Table 1:** Sensitivity testing of Citrobacter sedlakii isolate MIC, minimum inhibitory concentration; R, resistant; I, intermediate; S, sensitive *14mm zone of inhibition by Kirby-Bauer testing.

Antibiotic	MIC Interpretation	MIC
Amikacin	R	>32
Amoxicillin-clavulanate	R	>16/8
Ampicillin	R	>16
Ampicillin-sulbactam	R	>16/8
Aztreonam	R	>16
Cefazolin	R	>16
Cefepime	R	>16
Cefoxitin	R	>16
Ceftazidime	R	>16
Ceftazidime-avibactam	R	
Ceftolozane-tazobactam	R	
Ceftriaxone	R	>32
Ciprofloxacin	R	>2
Colistin	S	*
Eravacycline	S	
Ertapenem	R	>2
Gentamicin	R	>8
Meropenem	R	>8
Meropenem-vaborbactam	R	>16/8
Minocycline	I	
Moxifloxacin	R	>4
Piperacillin-tazobactam	R	>64/4
Tetracycline	R	>8
Tigecycline	S	≤1
Tobramycin	R	>8
Trimethoprim-sulfamethoxazole	R	>2/38

**Figure 1:**
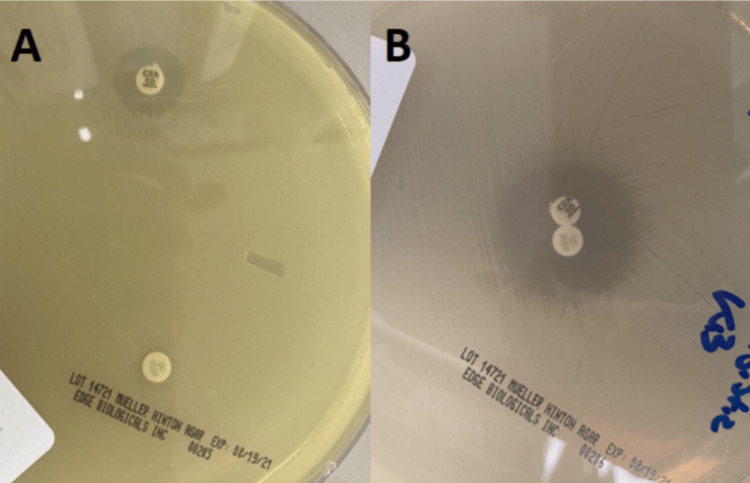
Susceptibility plating (A) separated ceftazidime-avibactam (top left) and aztreonam (bottom left); (B) combination plating

At this point, the patient was started on ceftazidime-avibactam and aztreonam (CAA) for salvage therapy. His CRE, *C. sedlakii*, appeared susceptible based on agar plating evaluating for synergy (Figure 1). This isolate was sent to the Tennessee Department of Health laboratory for confirmatory testing, which identified the presence of the New Delhi metallo-β-lactamase (NDM) using Streck ARM-D® (Streck, Nebraska, United States) reverse transcription-polymerase chain reaction (RT-PCR) testing. He stayed in the hospital to complete his six-week course of combination therapy. He tolerated this treatment well with no major complications, and there were no signs of residual infection after six weeks of CAA.

Two months after his treatment, he presented again draining purulence from his surgical site. He required surgical intervention including removal of a spacer, placement of a bone graft, and wound coverage. Surgical cultures at that time grew methicillin-sensitive *Staphylococcus aureus* but did not grow any other organism. He was able to be quickly de-escalated to minocycline for completion of treatment. He was then noted to be doing well without evidence of recurrence at a follow-up clinic appointment two weeks later.

## Discussion

Our case highlights the limited therapeutic options for the treatment of osteomyelitis due to MDR organisms and CREs. There is growing evidence that the combination of CAA is an effective therapeutic option for CRE infections, including those due to class B MBL. Aztreonam, a monobactam, has been shown to be effective against gram-negative organisms via the penicillin-binding protein 3 (PBP3) protein like other β-lactams, but unlike other β-lactams, is not hydrolyzed by class B MBL [4,5]. Avibactam is a β-lactamase inhibitor currently only commercially available in combination with ceftazidime and is typically active against Ambler class A, C, and D β-lactamases, which are commonly co-harbored by organisms producing MBL [5]. There is good rationale that CAA combination therapy might be effective in treating these MDR organisms.

Multiple studies have shown in vitro synergy between ceftazidime-avibactam and aztreonam against MBL-producing gram-negative pathogens. Despite the majority of these organisms being resistant to aztreonam and ceftazidime-avibactam individually, significantly lower minimum inhibitory concentrations associated with increased rates of susceptibility were seen with the CAA combination [4-8]. These findings have been supported by animal model data suggesting possible in vivo clinical efficacy as well [9].

There is growing evidence that CAA combination therapy is effective in the clinical setting as well. Numerous case studies involving MBL-carrying gram-negative organisms have displayed successful treatment with the aztreonam and avibactam combination [9-12]. A larger study of 102 patients with bloodstream infections with MBL-producing organisms (82 of which harbored NDM) showed decreased 30-day mortality, clinical failure at day 14, and length of stay in patients treated with aztreonam and ceftazidime-avibactam compared to other antibiotics [13]. Osteomyelitis, however, presents another challenge as it is often difficult to treat and requires a lengthy course of antibiotics, and data on the efficacy of aztreonam and avibactam to treat MBL osteomyelitis are more limited.

The data on MBL-associated osteomyelitis is limited to a handful of case reports. A regimen of aztreonam (2 g every eight hours) and ceftazidime-avibactam (2.5 g every eight hours) was used for a six-week treatment course in these cases with successful outcomes [9-11]. Our case adds to this growing body of work by providing another example of a case of osteomyelitis successfully treated with CAA. Our patient’s case was complicated by the fact that he continued to have hardware present, raising the concern for biofilm formation and increased fears of recurrence [14]. Despite this risk, our patient was able to tolerate CAA for the full six-week course and have resolution of his infection.

## Conclusions

In summary, MDR organisms and specifically MBL-producing organisms are a serious health threat with high mortality, morbidity, and economic burden. In addition to antimicrobial stewardship, there is a need to continue exploring new antibiotic regimens that can be used to fight these increasingly resilient organisms. We presented the case of an osteomyelitis caused by an MBL-producing *C. sedlakii* that was successfully treated with ceftazidime-avibactam and aztreonam. While we hope that this combination rarely needs to be used, we recommend consideration of it for infections caused by MBL-harboring organisms.
